# Is Raw Milk Injurious?

**Published:** 1901-07-06

**Authors:** 


### IS RAW MILK INJURIOUS?

The importance of securing a supply of milk free from taint of tuberculosis has been made light of by some in consequence of the ease with which it can be sterilised by boiling, for the investigations made by the Royal Commission on Tuberculosis have gone to show that the mere raising of the milk to the boiling point is sufficient to ensure the destruction of any tubercle bacilli which it may contain. But if we are to accept the dictum of those who maintain that the nutritive value of milk as a food is injured by boiling, then we come back to the necessity of securing its purity by careful attention to the conditions under which it is produced and distributed. At the same time, to be perfectly honest in the matter, it is to be noted that while definite proof of the conveyance of tuberculosis by milk can rarely be obtained, there is a good deal of experience which tends to show that the danger of tuberculosis from the ingestion of milk is not so great as some have supposed. Some time ago the late Dr. Kanthack showed how very large a proportion of the milk sold in Cambridge was contaminated with tubercle bacilli, to which the retort was at once made that if so these bacilli certainly did not appear to be doing any great harm among the students by whom the milk was being consumed. Among those who have protested most strongly against the boiling of milk is Dr. Clement Dukes, of Rugby, and his experience of the innocuousness of unboiled milk is a matter of some importance. He says[1]:—"That 'fresh' or raw milk cannot be so deleterious an article of food as is asserted is proved beyond all question by the fact that during a period of 30 years in which I have had charge of the health of a very susceptible community—a great public school—I have had only four cases of typhoid fever, three cases of diphtheria (not one of which was occasioned by milk), and none of tuberculous disease, and yet the boys drink an enormous quantity of milk—so great that I have been censured by a member of the profession for the quantity I advocate and have secured for their welfare, which is never boiled and rarely warmed." Here, then, we come to a very important problem. Either Dr. Dukes' boys have been extraordinarily lucky in their milk supply—and we must remember that thirty years goes back to before the time when we began to think so much of bovine tuberculosis as we do at present—or else milk infection cannot be so powerful and constantly acting a cause of tuberculosis as some have held it to be. We are all for cleanliness and purity in food supply; but all exaggeration is to be avoided, and such a statement as that made by Dr. Clement Dukes deserves the most serious consideration. He holds that the destruction of the nutritive qualities of milk by boiling, acting as it does constantly and upon large numbers, is a far greater evil than the occasional occurrence of diseases which can be caused by milk. It is, however, the illustration which he gives of the rarity of milk-produced disease among boys consuming a large quantity of raw milk that is at the present moment of the greatest interest.

[1] Lancet, June 29.

